# Diseases of Swine

**Published:** 1897-12

**Authors:** 


### REVIEW.

---

DISEASES OF SWINE. By D. McINTOSH, V.S., Professor of Veterinary Science in the University of Illinois.

THIS book has aimed to cover a field of comparative pathology very imperfectly understood, and to do it alike for the veterinary student and the swine-grower. It is only too true that much has been written as the result of original investigation by several American authorities. Still, of what has been studied there remain the widest divergence of opinion and admitted uncertainty of scientific, practical methods of dealing with these diseases. The obscurity in which a large number of swine-diseases still remain makes difficult the task of preparing a text-book of merit. To afford a work equally valuable to veterinarians, students, and swine-growers at once arouses one's fears that in the present light this would be an almost impossible task, and indeed it is very questionable whether efforts in this direction at the present day may not be more injurious than beneficial. A compilation of what has been the result of observation only, and based upon no accurate study, is not destined to aid the intelligent and progressive veterinarian. Doubtful or wholly empirical knowledge is dangerous in the hands of students or laymen, and is sure in the end to lead to a lessened appreciation of the profession. It is hoped, however, that this effort of the author will demonstrate the great commercial need of more extended knowledge, and thus lead to the production of a work abreast with the demands in this field.

---

A Vermont creamery makes 10,000 pounds of butter daily.

